# Flexible versus rigid endoscopy in the management of esophageal foreign body impaction: systematic review and meta-analysis

**DOI:** 10.1186/s13017-018-0203-4

**Published:** 2018-09-12

**Authors:** Davide Ferrari, Alberto Aiolfi, Gianluca Bonitta, Carlo Galdino Riva, Emanuele Rausa, Stefano Siboni, Francesco Toti, Luigi Bonavina

**Affiliations:** Department of Biomedical Sciences for Health, Division of General Surgery, IRCCS Policlinico San Donato, University of Milan, Piazza E. Malan, 1, 20097 San Donato Milanese, Milan, Italy

**Keywords:** Esophageal foreign body, Flexible endoscopy, Rigid endoscopy, Iatrogenic esophageal perforation, Foreign body impaction

## Abstract

**Background:**

Foreign body (FB) impaction accounts for 4% of emergency endoscopies in clinical practice. Flexible endoscopy (FE) is recommended as the first-line therapeutic option because it can be performed under sedation, is cost-effective, and is well tolerated. Rigid endoscopy (RE) under general anesthesia is less used but may be advantageous in some circumstances. The aim of the study was to compare the efficacy and safety of FE and RE in esophageal FB removal.

**Methods:**

PubMed, MEDLINE, Embase, and Cochrane databases were consulted matching the terms “Rigid endoscopy AND Flexible endoscopy AND foreign bod*”. Pooled effect measures were calculated using an inverse-variance weighted or Mantel-Haenszel in random effects meta-analysis. Heterogeneity was evaluated using *I*^2^ index and Cochrane *Q* test.

**Results:**

Five observational cohort studies, published between 1993 and 2015, matched the inclusion criteria. One thousand four hundred and two patients were included; FE was performed in 736 patients and RE in 666. Overall, 101 (7.2%) complications occurred. The most frequent complications were mucosal erosion (26.7%), mucosal edema (18.8%), and iatrogenic esophageal perforations (10.9%). Compared to FE, the estimated RE pooled success OR was 1.00 (95% CI 0.48–2.06; *p* = 1.00). The pooled OR of iatrogenic perforation, other complications, and overall complications were 2.87 (95% CI 0.96–8.61; *p* = 0.06), 1.09 (95% CI 0.38–3.18; *p* = 0.87), and 1.50 (95% CI 0.53–4.25; *p* = 0.44), respectively. There was no mortality.

**Conclusions:**

FE and RE are equally safe and effective for the removal of esophageal FB. To provide a tailored or crossover approach, patients should be managed in multidisciplinary centers where expertise in RE is also available. Formal training and certification in RE should probably be re-evaluated.

## Background

Foreign body (FB) impaction accounts for 4% of all emergency endoscopies in clinical practice [1, 2], with 60% of adult patients being treated for food bolus [3]. Foreign body ingestion is a common occurrence in the USA, with more than 100,000 reported cases each year [4], and it is estimated that 1500 people die annually. [5] Foreign body-induced perforations represent 12% of all esophageal perforations and carry a 2.1% mortality [6]. Esophageal FB impaction in adults is commonly associated with underlying esophageal disease [7–10] or psychiatric disorders [11–14].

In a retrospective study, the location of impacted FB was the cervical esophagus in 57% of cases, thoracic esophagus in 26%, and esophago-gastric junction in 17% [15]. Physiologically, the transition from striated skeletal muscle to smooth muscle explains why the upper esophagus is the most common site of impaction. Fifty percent of sharp objects tend to lodge in the upper esophagus and frequently cause perforation, especially after multiple attempts to endoscopic retrieval. Eventually, rigid endoscopy or surgery by cervical esophagotomy/thoracotomy may be required [16].

Nowadays, endoscopy is generally recommended as the first-line therapeutic option, [17, 18] whereas surgery is considered as a suitable upfront treatment in patients presenting with overt perforation or rescue treatment in case of irretrievable FB [19–22]. Flexible endoscopy (FE) can be performed under local anesthesia and sedation and is cost-effective since it does not require hospitalization [23–25]; however, its effectiveness is limited in case of sharp FB impaction [15, 26].

Rigid endoscopy (RE) provides a wide operating lumen, which gives a great advantage in the manipulation of sharp FB impacted in the upper esophagus; in addition, it allows the extraction of FB with multiple instruments, and the airways are protected because the procedure is performed under general anesthesia. Interestingly, the skills for performing rigid endoscopy are limited among non-ear-nose-throat (ENT) specialists to the point that RE is not even mentioned in the most recent European guidelines [18].

Should RE be totally abandoned or still has a role? The aim of this systematic review and meta-analysis was to assess the efficacy and complications of RE and FE in removing esophageal FB.

## Materials and methods

We conducted this study according to the Preferred Reporting Items For Systematic Reviews and Meta-Analyses (PRISMA) statement. An extensive literature search was conducted by two independent authors to identify the English-written published series on studies comparing RE and FE in the management of esophageal FB. PubMed, MEDLINE, Embase, and Cochrane databases were consulted matching the terms “Rigid endoscopy AND Flexible endoscopy AND foreign bod*”. The search was completed by consulting the listed references of each article. Studies were included if success rates of flexible and rigid endoscopy were described in the extraction of foreign bodies impacted in the esophagus. Both studies on adult and pediatric populations were included. Studies were excluded if no distinction was made between the FE group and the RE group or if the methodologies were compared outside the treatment of FB impaction.

Two authors (AA, DF) independently read the 200 abstracts generated by the literature search, and 15 duplicates were removed. Among the 185 records screened, 178 abstracts were excluded as they did not meet inclusion criteria. Seven abstracts assessed for eligibility were reviewed in full-text. Two more articles were excluded, one because it focused on the diagnosis of esophageal disease and the other because of missing data.

**Fig. 1:**
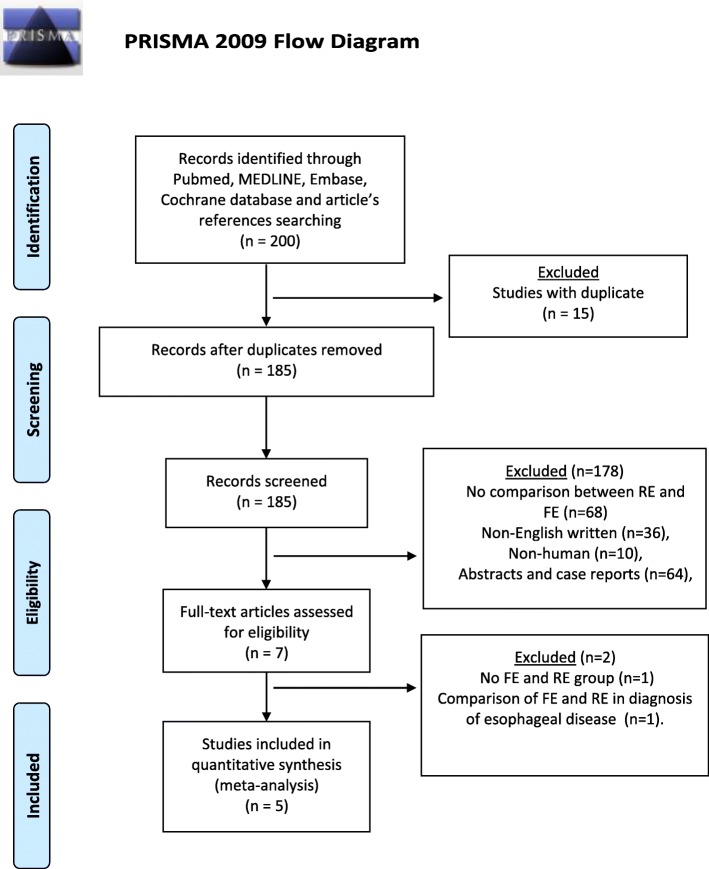
PRISMA diagram

All articles comparing RE and FE in foreign body management were included in the systematic review (Fig. 1). Three authors (DF, AA, CGR) independently extracted data from eligible studies. Data extracted included study characteristics (first author name, year, and journal of publication), number of patients included in the series, localization of foreign bodies, characteristics of the FB, endoscopic success rate, overall complication rate, and perforation rate. Endoscopy was considered successful if the FB was removed. Endoscopy was not considered successful if conversion to another endoscopic technique or surgery was needed. Disagreements between authors were resolved by consensus; if no agreement could be reached, a fourth senior author (LB) made the decision. Three investigators independently assessed the methodological quality of the papers using the Newcastle-Ottawa Scale (NOS) [27]. Each study is judged on a “star system” based on the selection of the study groups and the ascertainment of the outcome of interest. Each study could earn a maximum of nine stars.

### Statistical analysis

The results of the systematic review were summarized qualitatively into a frequentist meta-analysis. For the pooled measure of effect size, an inverse-variance weighted or Mantel-Haenszel random effects meta-analysis was performed, as appropriate. The DerSimonian-Laird estimator was used to estimate between-study variance (*τ*^2^) [28]. Zero cell count are accounted according to Yusuf et al. [29]. Heterogeneity among the studies was evaluated by *I*^2^ index and Cochran *Q* test [30]. Statistical heterogeneity was considered significant when *p* < 0.10 or *I*^2^ index was > 50% [31]. Wald-type 95% confidence intervals were computed for the pooled measure; otherwise, 95% confidence intervals for *I*^2^ index were calculated according to Higgins and Thompson [32]. Small study and publication bias effects were assessed by *trim and fill* method [33]. Egger tests were applied [34]. The prediction interval for the treatment effect of a new study is calculated according to Borenstein [30]. As the sample size is not the same in all studies, we performed a sensitivity analysis by excluding one study each time and rerunning the analysis to verify the robustness of the overall results. *Z*-score test was performed. Two-sided *p* value was considered statistically significant when < 0.05. All analyses and figures were carried out using R version 3.2.2 software [35].

## Results

### Systematic review

Five studies published between 1993 and 2015 matched the inclusion criteria. The total number of patients was 1402; the sample size of the individual studies ranged from 118 to 657. There were no randomized controlled studies. All reports were observational cohort studies. Each study reached a NOS score of 6 or 7 (median 6.9), suggesting a good quality level.

Demographic, clinical, and operative variables of the patient sample are shown in Table 1. All patients underwent endoscopy for the removal of FB impacted in the esophagus. Overall, 736 patients underwent FE under local anesthesia and sedation, and 666 underwent RE under general anesthesia. The mean age of the patients ranged from 3.5 to 64 years, and more than half of them were females.

**Table 1:** Demographic and clinical data of 1402 patients undergoing rigid endoscopy (RE) or flexible endoscopy (FE)

Author, year, country	Study design	#Pts	Mean age	Male (%)	Procedure	Foreign body type (*n*)				Procedures attempted (*n*)	Successful procedures (*n*)	Overall complications (*n*)	Perforations (*n*)	Esophageal localization (*n*)		
						Blunt	Sharp	Long	Food					Upper	Middle	Lower
Bergreen, 1993, USA	Retrospective	118	31.5	nr	FE	22	1	0	51	78	75	4	0	25	6	46
					RE	35	1	0	4	40	40	4	0	28	5	3
Gmeiner, 2007, Austria	Retrospective	138	64	49.6	FE	9	0	0	62	76	71	0	0	18	11	45
					RE	13	0	46	0	62	59	2	2	54	3	1
Russell, 2014, USA	Retrospective	657	3.5	55	FE	nr	nr	nr	nr	366	359	5	0	290	36	34
					RE					291	281	6	0	220	28	41
Tseng, 2015, Taiwan	Retrospective	273	48.7	41.4	FE	nr	nr	nr	nr	142	137	1	1	nr	nr	nr
					RE					131	126	5	3	nr	nr	nr
Wang, 2014, China	Retrospective	216	50.6	42.0	FE	14	38	0	22	74	72	34	1	45	21	8
					RE	53	84	0	5	142	142	40	8	126	13	3

*nr* not reported

By intention to treat, FE was successful in 714 (97.0%) patients and RE in 648 (97.3%). The type of FB ingested, classified in accordance with the latest guidelines [18], were reported in three studies (472 patients). Food bolus was the most frequent event (*n* = 190, 41.3%), and the majority (71%) of these patients were treated with FE. Blunt objects and sharp-pointed objects accounted for 31.7% and 27.0%, respectively; the latter were mainly approached with RE (*n* = 85, 68.5%). The site of esophageal impaction was reported in four studies (1110 patients). Overall, 806 patients (72.6%) had upper esophageal FB impaction; of these, 378 (46.9%) were managed with FE and 428 (53.1%) with RE. There were 123 impactions in the middle esophagus (11.1%); 74 patients (60.2%) were managed with FE and 49 (39.8%) with RE. Lastly, 181 patients (16.3%) had lower esophageal impaction and the majority were treated with FE (*n* = 133, 73.5%).

The overall complication rate was 7.2%. The most frequently reported complications were mucosal erosion (*n* = 27, 26.7%), mucosal edema (*n* = 19, 18.8%), and iatrogenic esophageal perforation (*n* = 15, 10.9%) (Table 2). There was no mortality.

**Table 2:** Complications during endoscopy

Complication	%	Total (*n*)
Erosion	26.7	27
Mucosal edema	18.8	19
Others	13.9	14
Perforation	10.9	11
Ulcer	10.9	11
Hemorrhage	8.9	9
Post-extraction dilation	5.0	5
Mucosa denudation	3.0	3
Infection	2.0	2

### Meta-analysis

In addition to a systematic review, we performed a frequentist random effect model meta-analysis including five studies with a total of 1402 patients.

**Fig. 2:**
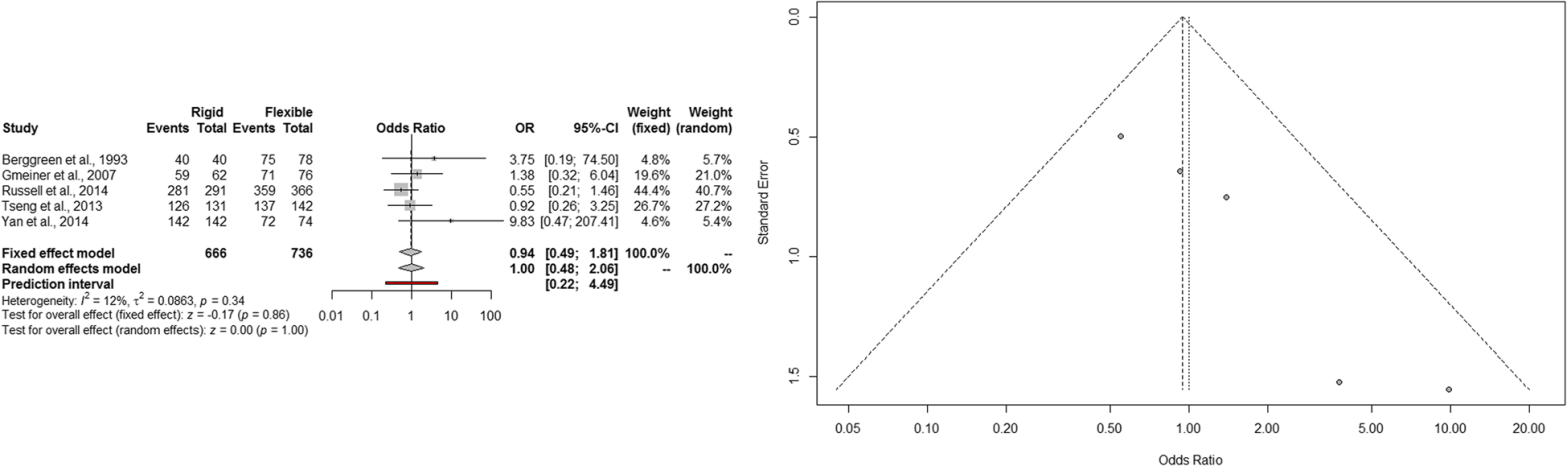
Forest and funnel plot of success rate

The estimated pooled odds ratio of procedure success is 1.00 (95% CI 0.48–2.06; *p* = 1.00). The prediction lower and upper limits are 0.22 and 4.49, respectively. The heterogeneity is not significant (*I*^2^ = 12%, 95% CI 0.0–81.7%; *p* = 0.34) and *τ*^2^ = 0.0863. Funnel plot shows that the publication and small study bias effect could not be rejected according to the Egger test (*p* < 0.001). The adjusted *trim and fill* odds ratio is 0.82 (95% CI 0.35–1.88) (Fig. 2). The sensitivity analysis shows the robustness of the results.

**Fig. 3:**
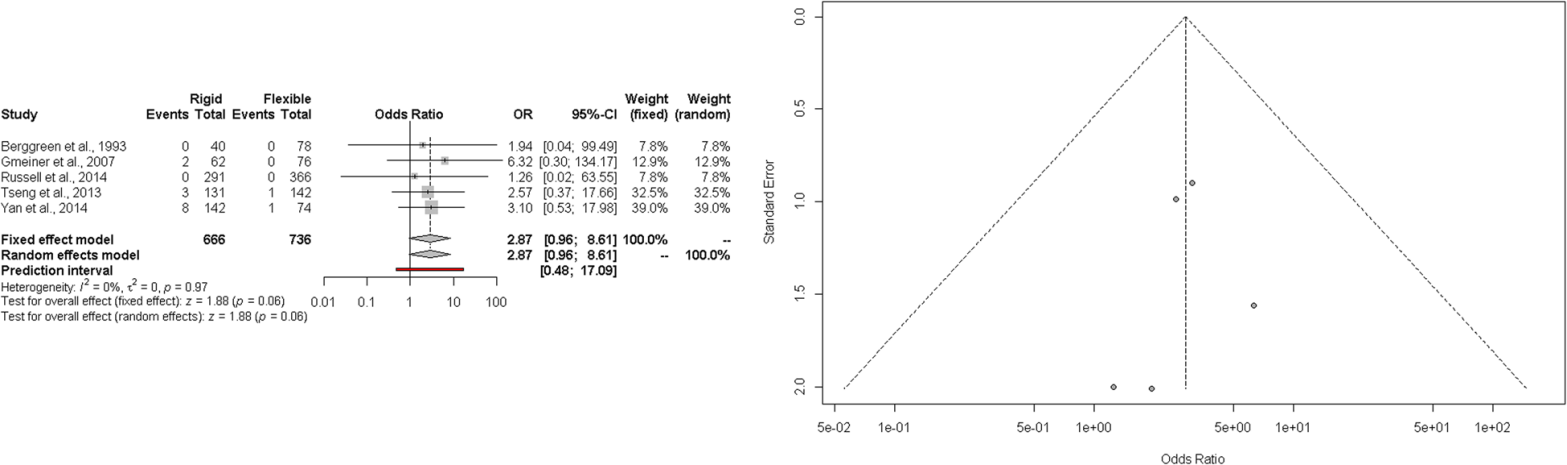
Forest and funnel plot of perforation rate

The estimated pooled odds ratio of perforation rate is 2.87 (95% CI 0.96–8.61; *p* = 0.06). The prediction lower and upper limits are 0.48 and 17.09, respectively. The heterogeneity is not significant (*I*^2^ = 0%, 95% CI 0.0–0.1%; *p* = 0.97) and *τ*^2^ = 0. Visual inspection of funnel plot shows that the publication and small study bias effect could not be rejected according to the Egger test (*p* = 0.32). The adjusted *trim and fill* odds ratio is 3.1 (95% CI 1.06–8.87) (Fig. 3). The sensitivity analysis shows the robustness of the results.

**Fig. 4:**
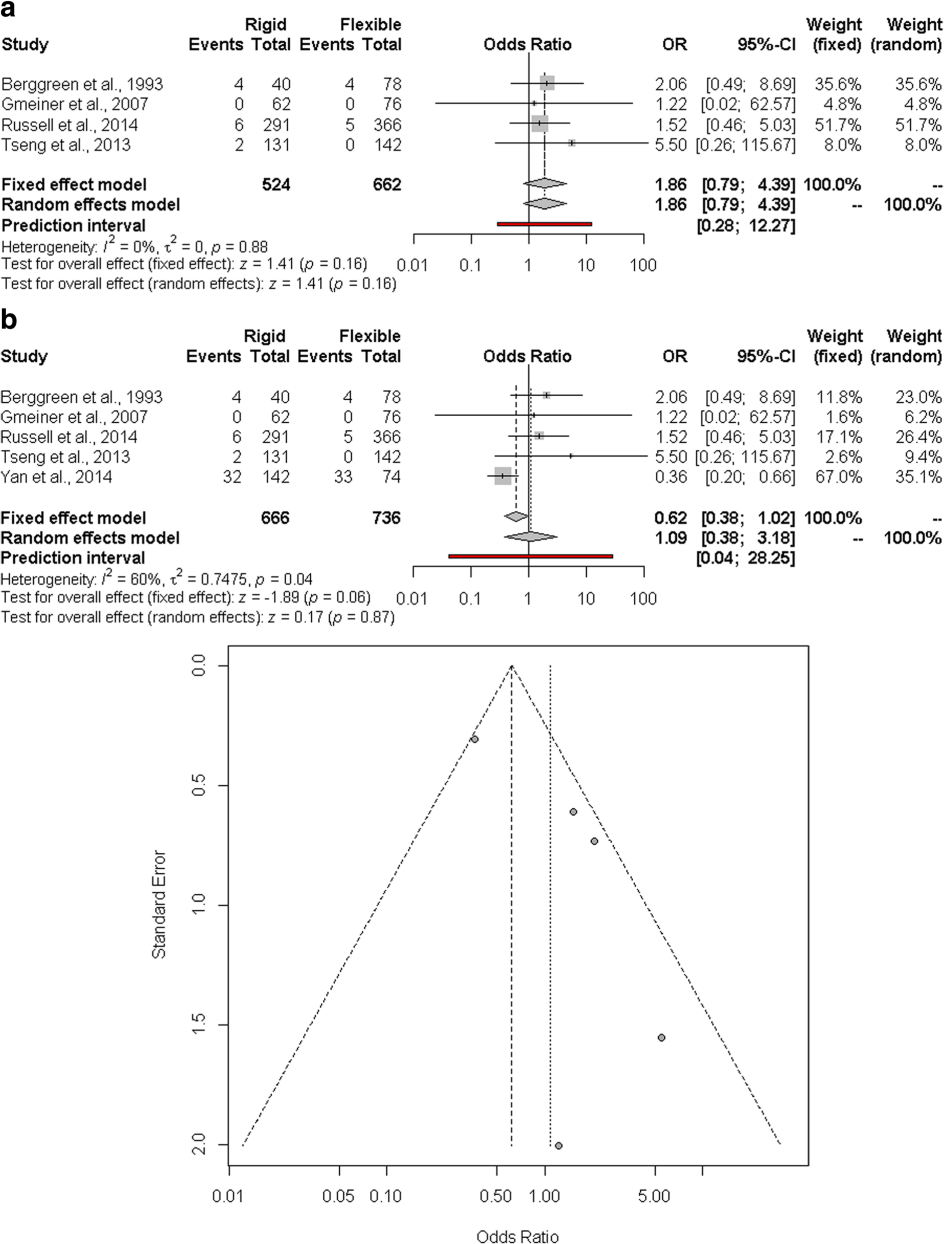
**a** Forest and funnel plot of other complications. **b**. Forest plot of other complications by omitting Wang

The estimated pooled odds ratio of other complications is 1.09 (95% CI 0.38–3.18; *p* = 0.87). The prediction lower and upper limits are 0.04 and 28.25, respectively. The heterogeneity is moderate (*I*^2^ = 60%, 95% CI 0.0–85.0%; *p* = 0.04) and *τ*^2^ = 0.7475. Funnel plot shows that the publication and small study bias effect could not be rejected according to the Egger test (*p* = 0.03). The adjusted *trim and fill* odds ratio is 0.40 (95% CI 0.14–1.14) (Fig. 4a). The sensitivity analysis showed that the results are affected by the study of Wang., in particular regarding heterogeneity. After excluding this study, the heterogeneity was decreased (*I*^2^ = 0.0%, 95% CI 0.0–0.3%), and the pooled odds ratio was 1.86 (95% CI 0.79–4.39; *p* = 0.16) (Fig. 4b).

**Fig. 5:**
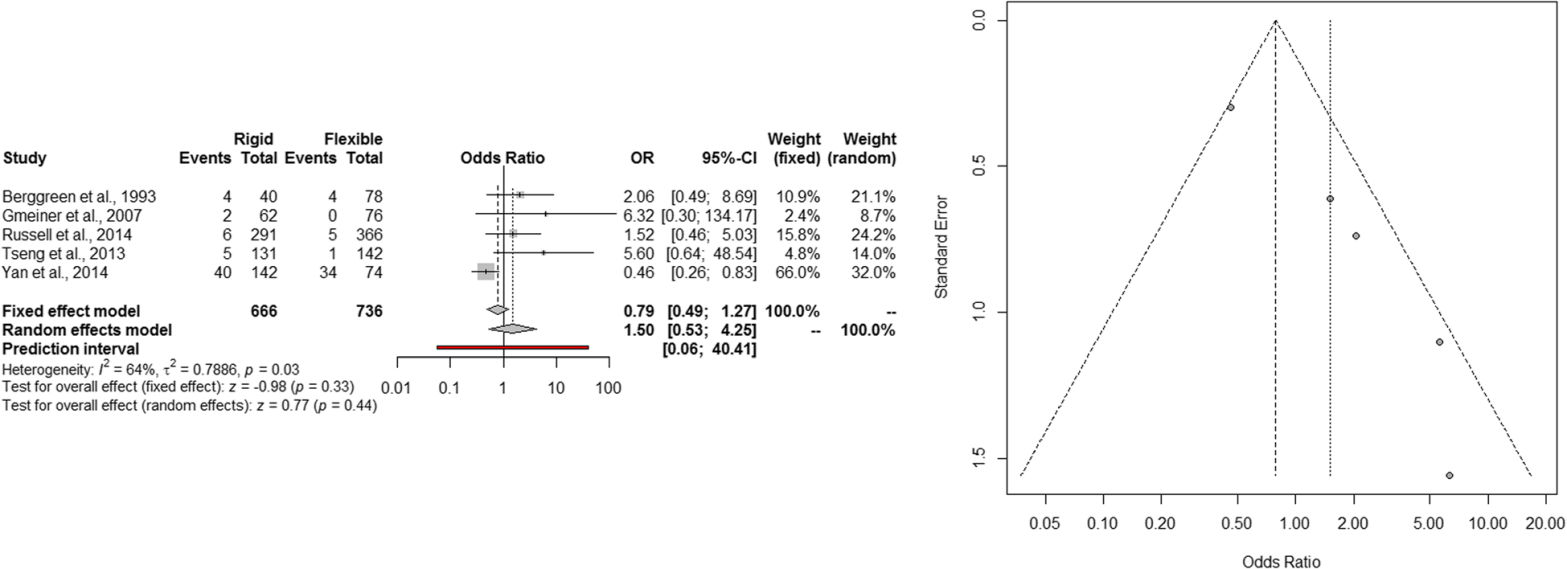
Forest and funnel plot of overall complications

The estimated pooled odds ratio of overall complications is 1.50 (95% CI 0.53–4.25; *p* = 0.44). The prediction lower and upper limits are 0.06 and 40.41, respectively. The heterogeneity is moderate (*I*^2^ = 64%, 95% CI 4.3–86.2%; *p* = 0.03) and *τ*^2^ = 0.7886. Visual inspection of funnel plot shows that the publication and small study bias effect could not be rejected according to the Egger test (*p* = 0.004). The adjusted *trim and fill* odds ratio is 0.64 (95% CI 0.24–1.72) (Fig. 5). The sensitivity analysis shows the robustness of the results.

## Discussion

This study shows that RE still plays an important therapeutic role in patients with upper esophageal FB impaction, especially in the case of ingested sharp-pointed objects or when general anesthesia is recommended (i.e, in children or in patients with concomitant respiratory symptoms). In case of a large blunt FB impaction, the excellent exposure of the upper esophagus provided by the rigid endoscope may be crucial for a safe and successful extraction.

In the 2011 American Society for Gastrointestinal Endoscopy guidelines, the decision to perform RE or FE was left to the clinician’s judgment [17]. Rigid esophagoscopy was considered helpful for proximal FB impacted at the upper esophageal sphincter. In contrast, in the most recent 2016 European guidelines, RE is not even mentioned [18]. This may be due to the lack of expertise of non-ENT specialists in performing RE [26] and to the fact that previous retrospective studies and expert opinions reported a theoretical superiority of FE over RE. Gmeiner et al. [25] reported higher patient comfort and lower complication rate with FE and proposed a crossover management (from FE to RE and vice versa) in case of failure of the initial treatment. Since FB impaction lasting longer than 24 h leads to a higher risk of perforation [1, 3, 36–40], a multidisciplinary approach in tertiary care centers, where both FE and RE are available, may represent the safest strategy in these patients [41].

The choice of the most suitable approach in the management of impacted esophageal FB depends on factors related to the patient (age, clinical condition, compliance, American Society of Anesthesiologists (ASA) score), type and size of FB, anatomical site of impaction, timing of impaction, and physicians’ expertise [18]. Surgery should be considered as an upfront treatment in patients with overt esophageal perforation or as a rescue treatment in endoscopically irretrievable esophageal FB. More recently, advances in minimally invasive surgery have allowed a thoracoscopic approach in selected patients [42]. Open or minimally invasive esophagectomy [43], with immediate or delayed reconstruction, should represent the ultimate surgical option. Since the burden of esophageal perforation remains high, especially in developing countries [26], re-evaluating the role of RE training and certification may contribute to an overall decrease of surgery-related morbidity and mortality in the future. The shift from open surgical to endo-surgical approach for the treatment of Zenker diverticulum in dedicated centers has led to a re-appraisal and increasing use of the Weerda diverticuloscope [44–46]; this instrument allows to introduce a video endoscope or a rigid 5-mm telescope through the operative channel along with multiple devices (linear endo-staplers, grasping forceps, etc.).

In our study, both FE and RE were effective and safe, and the success and overall complication rates were similar (Table 2). Although the estimated pooled perforation rate odds ratio (OR) was not significant (*p* = 0.06), the point estimation for RE was 2.87, thus suggesting a possible clinical relevance. This may be related to the fact that RE, especially in adults, is generally used as second-line therapy after FE failure or as first-line therapy in more challenging situations (i.e., sharp-pointed or large foreign bodies) [16]. In a pediatric population, Russell et al. [47] reported no perforations in the RE group [47]. This is interesting since children with FB impaction often undergo upfront RE under general anesthesia due to low compliance and need to protect the airways.

Although we included studies reporting on both pediatric and adult populations, the sensitivity analysis showed low heterogeneity. Furthermore, excluding the article by Wang. [48], there was no heterogeneity (*I*^2^ = 0.0%, *τ*^2^ = 0.0%). This indicates that minor complications, such as mucosal edema and erosions, were not considered relevant in other studies.

This meta-analysis has some limitations. Only retrospective comparative studies were included since, due to the nature of the topic, no randomized clinical trials or prospective studies were available. However, the average quality of the included studies was good. In addition, timing to endoscopy, patients’ ASA scores and the rationale for choosing FE or RE as an initial therapeutic option were not reported.

## Conclusions

In conclusion, FE and RE appear to be equally effective for the removal of esophageal FB, and differences in overall complication and perforation rates are not statistically significant. In selected cases, the two methods may be complementary; therefore, patients should be managed in centers where expertise in RE is also available to allow a tailored or cross-over approach, with the aim to reduce the need for surgery and related morbidity. Formal training and certification in RE should probably be re-evaluated.

## Abbreviations

ASA: American Society of Anesthesiologists
ENT: Ear-nose-throat
FB: Foreign bodies
FE: Flexible endoscopy
N: Number
OR: Odds ratio
Pts: Patients
RE: Rigid endoscopy
